# No Female Germline Stem Cells Were Identified by Advanced Dual Recombinase‐Based Lineage Tracing System

**DOI:** 10.1111/cpr.70289

**Published:** 2026-10-20

**Authors:** Yang Gao, Zhiming Shen, Xiuhong Cui, Min Chen, Mengyue Wang, Kui Liu, Fei Gao, Hongmei Wang

**Affiliations:** ^1^ State Key Laboratory of Organ Regeneration and Reconstruction, Institute of Zoology, Chinese Academy of Sciences Beijing China; ^2^ University of Chinese Academy of Sciences Beijing China; ^3^ Research Institute for Marine Traditional Chinese Medicine (Qingdao Academy of Chinese Medical Sciences), the SATCM'S Key Unit of Discovering and Developing New Marine TCM Drugs Key Laboratory of Marine Traditional Chinese Medicine in Shandong Universities, Shandong University of Traditional Chinese Medicine Jinan China; ^4^ Shandong University of Traditional Chinese Medicine Qingdao Academy of Chinese Medical Sciences, Qingdao Key Laboratory of Research in Marine Traditional Chinese Medicine, Qingdao Key Technology Innovation Center of Marine Traditional Chinese Medicine's Deep Development and Industrialization Qingdao China; ^5^ Beijing Institute for Stem Cell and Regenerative Medicine Beijing China; ^6^ State Key Laboratory of Reproductive Medicine and Offspring Health, Center for Reproductive Medicine, Institute of Women, Children and Reproductive Health, Shandong University Jinan China; ^7^ Department of Obstetrics and Gynecology, Li Ka Shing Faculty of Medicine The University of Hong Kong Hong Kong China; ^8^ Shenzhen Key Laboratory of Fertility Regulation, Center of Assisted Reproduction and Embryology, The University of Hong Kong‐Shenzhen Hospital Shenzhen Guangdong China

**Keywords:** Dual recombinase system, Female germline stem cells, Lineage tracing, *Stra8*

## Abstract

Whether female germline stem cells (fGSCs) exist in postnatal mammalian ovaries has been debated for decades. Since the fGSCs were first reported in 2004, a large number of studies have been conducted using different approaches. However, the persistent controversy surrounding the results has caused confusion among many researchers and clinicians. To clearly clarify this question, we developed advanced tracing system to label any possible fGSCs in adult female mice. Our results showed that no Stra8‐expressing germ cells were detected in the ovaries of adult female mice using a long‐term uninterrupted lineage tracing system under both physiological and pathological conditions. Furthermore, we adopted the interleaved reporter tracing system that allows mutually exclusive labelling of germ cells and non‐germ cell populations while preventing potential secondary recombination. This approach revealed no evidence of *de novo* germ cell formation during postnatal life. Together, our findings provide the evidence that fGSCs and postnatal oogenesis do not exist under both physiological and pathological conditions in mouse models, therefore reinforcing the notion that the primordial follicle pool established perinatally serves as the primary source of oocytes through the entire reproductive lifespan.

## Graphical abstract

Schematic summary of the dual‐recombinase lineage tracing strategies to determine whether female germline stem cells (fGSCs) exist and sustain the ovarian follicle pool in adult female mice. Our study concluded that no fGSCs exist and contribute to oocyte regeneration in adult female mice with different tracing systems.

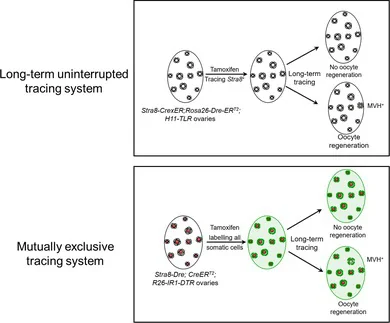

## 1 Introduction

In mammals, the ovaries are responsible for producing fertilizable oocytes and secreting the hormones and factors that maintain female reproductive characteristics [1, 2]. Ovarian follicles are the basic functional units which are formed perinatally via cyst breakdown [3, 4]. The primordial follicles are composed of one oocyte surrounded by a single layer of squamous pre‐granulosa cells [5]. It has been believed that the number of primordial follicles is determined once they are formed and will not be replenished throughout the entire reproductive lifespan [6, 7]. However, the debate over the existence of female germline stem cells (fGSCs) was started in 2004, and more than 80 studies have been published by different research groups on this topic. The existence of fGSCs has been characterised in multiple species, including mice [8, 9, 10], rats [11], pigs [12], cows [13], non‐human primates [14] and humans [8, 15]. Although the debate over the existence of fGSCs has lasted for decades, the evidence remains controversial. In 2004, Jonathan Tilly's group first reported mitotically active germ cells in the ovarian surface epithelium, together with the statistical analysis, they proposed that fGSCs exist in postnatal mouse ovaries [16]. Furthermore, they proposed that putative fGSCs reside in bone marrow and sustain oocyte production after birth under both physiological and pathological conditions [17]. Both Wu's lab and Jonathan Tilly's group isolated fGSCs using anti‐DDX4 antibody and demonstrated their ability to develop into oocytes in vivo [10, 15]. Wu's group further reported that these cells are capable of generating offspring [10]. The following study by Zheng's lab identified a group of Oct4‐positive putative fGSCs in the ovarian cortex of adult mice and reported that these cells underwent meiosis and formed primordial follicles [18]. In 2017, *Stra8*‐expressing germ cells were detected in mouse ovaries after birth using lineage tracing mouse model and these cells were reported to form functional oocytes [19].

However, studies from other research groups have reached completely opposite conclusions. The studies using parabiosis models refuted the bone marrow origin hypothesis and found no evidence that the putative fGSCs contribute to new oocytes [20], while  statistical analyses demonstrated that the primordial follicle pool established perinatally is sufficient to sustain the oocyte population required for fertility throughout the reproductive lifespan [21]. The experimental approach of isolating putative fGSCs was subsequently challenged by Zhang et al., who raised concerns regarding the reliability of DDX4 antibody‐based isolation, as DDX4 was reported to be localised in the cytoplasm of ovarian germ cells [22]. The studies using cell lineage tracing with the Cre‐loxP recombination system by Zhang et al. and Lei et al. also reported that no mitotically active germ cells exist in adult ovaries and no postnatal folliculogenesis was detected [23, 24, 25]. A recent study using high‐throughput single‐cell sequencing did not identify fGSCs in the human ovarian cortex [26]. However, both conventional lineage‐tracing and single‐cell sequencing approaches have limitations in detecting rare fGSCs; therefore further investigation is needed to clarify this question.

To further clarify and hopefully resolve this long‐term debate, we introduced the dual recombinase‐based lineage tracing system, which is based on the specific recombination of Cre‐loxP [27] and Dre‐rox [28]. As a more powerful tool, the dual recombinase‐based lineage tracing system allows a minimum interference of unintended labelling while ensuring accurate activation in the appropriate cell type at designated time points [29], and has enabled researchers to advance our understanding in several critical biological processes, including beta‐cell development [30], hepatocyte generation [31] and the origin of alveolar stem cells [32]. *Stra8*, the gatekeeper of meiosis initiation, is exclusively expressed in germ cells during meiosis initiation. In mouse model, *Stra8* expression begins in female germ cells at E12.5 and disappears around E15.5. As a gatekeeper of meiosis initiation, inactivation of *Stra8* causes the detect of mitosis‐to‐meiosis transition in germ cells [33, 34, 35].

Considering the essential role of *Stra8* in meiosis initiation, we hypothesised that if fGSCs existed and generated the functional oocytes, they should undergo meiosis initiation accompanied with *Stra8* expression. Therefore, we developed the dual recombinase‐mediated lineage tracing system to continuously and seamlessly record *Stra8‐*expressed meiosis initiation events in adult ovaries. With this dual lineage tracing strategy, no evidence of meiosis initiation was detected during long‐term tracing of 3 and 6 months in postnatal ovaries under both physiological and pathological conditions. Meanwhile, to further investigate whether other cell types can develop into oocytes regardless of whether they underwent meiosis, we used the interleaved reporter mouse model (*R26‐IR1*) [30, 36] to separately label germ cells and non‐germ cell populations with different markers. Our findings indicate that non‐germ cells do not develop into oocytes in postnatal ovaries under both physiological and pathological conditions. Thus, these findings provide supporting evidence that no fGSCs contribute to the generation of functional oocytes in adult mice.

## 2 Results

### 2.1 No Stra8‐Expressing Germ Cells Were Detected in Postnatal Ovaries

To investigate whether fGSCs exist in ovaries and initiate meiosis after birth, a *Stra8‐CreER* ^*T2*^ mouse model was generated and the knock‐in strategy was illustrated as in Figure S1A. Genotyping of *Stra8‐CreER* ^*T2*^ was shown in Figure S1B. In *Stra8‐CreER* ^*T2*^; *mT/mG* mice, the meiotic germ cells would be labelled with GFP after Tamoxifen treatment. To validate the mouse model, the expression of GFP in *Stra8‐CreER* ^*T2*^; *mT/mG* mice was analysed at different developmental stages with Tamoxifen treatment (Figure S2A,C,E). As shown in Figure S2B, GFP signal was detected in germ cells of female *Stra8‐CreER* ^*T2*^; *mT/mG* mice with Tamoxifen treatment at E12.5. By contrast, no GFP signal was detected in germ cells of female *Stra8‐CreER* ^*T2*^; *mT/mG* mice at P7 with or without Tamoxifen treatment at E15.5 (Figure S2C,D, arrowheads). To further verify this mouse model, 4‐week‐old male and female *Stra8‐CreER* ^*T2*^; *mT/mG* mice were injected with Tamoxifen and the expression of GFP was analysed 2 days later. GFP signal was detected in germ cells of *Stra8‐CreER* ^*T2*^; *mT/mG* testis after Tamoxifen treatment, while no GFP signal was detected in ovaries of *Stra8‐CreER* ^*T2*^; *mT/mG* mice (Figure S2E,F). These results indicated that the *Stra8‐CreER* ^*T2*^ mouse model was successfully generated and *Stra8‐CreER* ^*T2*^ could be specifically induced by Tamoxifen in pre‐meiotic germ cells.

To test whether fGSCs exist and initiate meiosis in adult ovaries, 4 weeks old *Stra8‐CreERT2; mT/mG* females were treated with Tamoxifen for six consecutive times and the ovaries were collected 1 week after treatment (Figure 1A). The results of immunostaining for GFP and MVH (also known as DDX4, a marker of germ cells) showed that all MVH positive germ cells were GFP negative (Figure 1B,C), indicating that no germ cells initiated meiosis in the ovaries of adult females. To further test whether fGSCs could initiate meiosis under pathological condition, we generated a POI (premature ovarian insufficiency) mouse model by treating mice with busulfan and cyclophosphamide as reported previously (Figure 1D) [17]. After chemotherapy treatment, the female *Stra8‐CreER* ^*T2*^; *mT/mG* mice were treated with Tamoxifen and the expression of GFP in ovaries was examined 2 weeks later (Figure 1D–F). The results of immunostaining showed that all MVH positive germ cells were GFP negative and no GFP positive oocyte was detected (Figure 1E,F, arrows).

**FIGURE 1:**
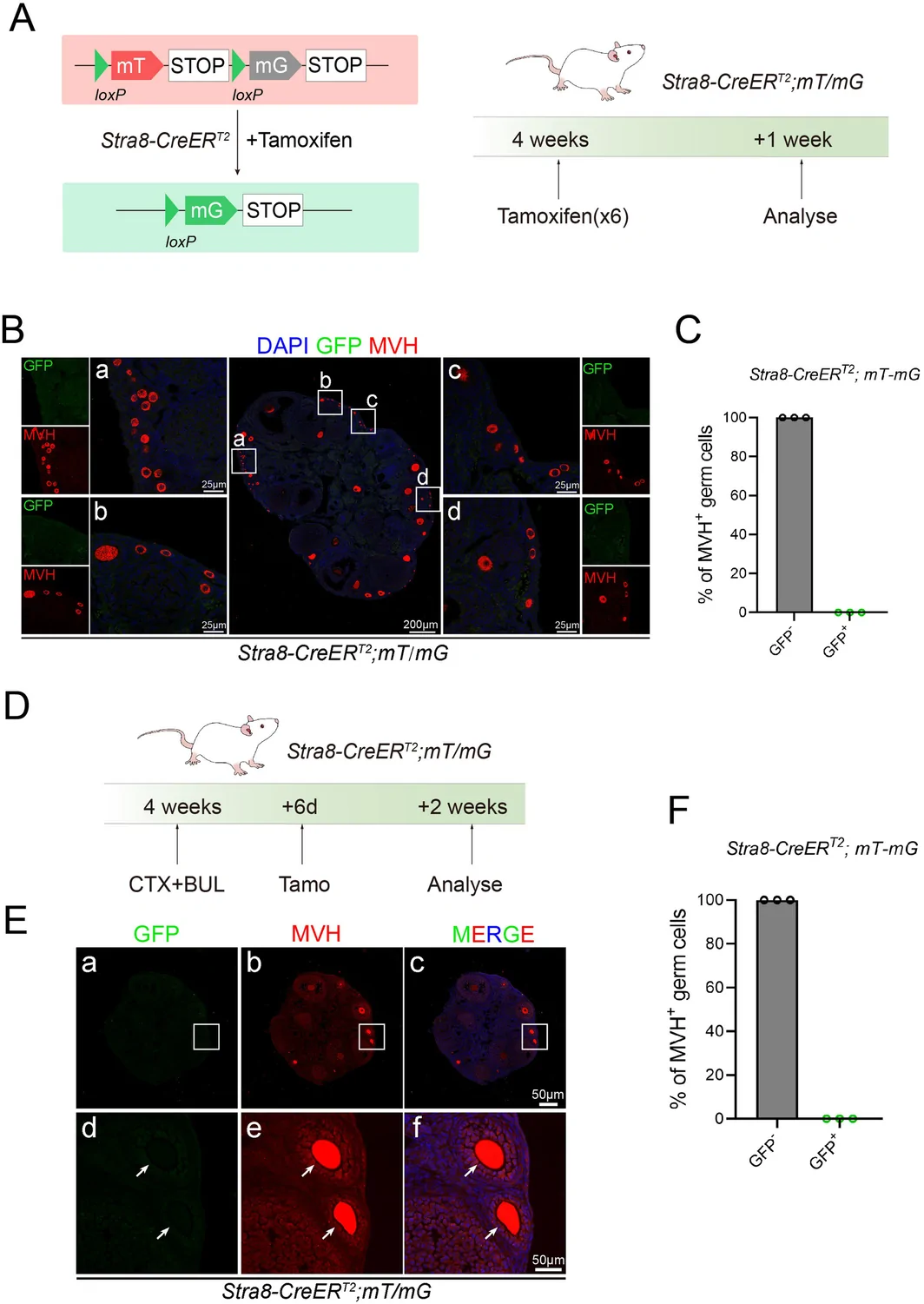
No Stra8‐expressing germ cells were detected in the ovaries of 4 weeks old female mice under both physiological and pathological conditions. (A) Schematic illustration of the experimental strategy to label the Stra8‐positive meiotic cells in 4 weeks old female mice. (B) The expression of GFP and MVH in the ovaries of *Stra8‐CreER* ^*T2*^ *; mT/mG* mice was examined by immunostaining. No GFP (green) and MVH (red) double‐positive germ cells were observed in the ovaries of *Stra8‐CreER* ^*T2*^ *; mT/mG* mice after six consecutive Tamoxifen treatments at 4 weeks. (C) Quantitative analysis of MVH and GFP double positive germ cells in the ovaries of *Stra8‐CreER* ^*T2*^ *; mT/mG* mice after Tamoxifen treatment at 4 weeks. (D) Schematic illustration of the experimental strategy to label the Stra8‐positive meiotic cells in oocyte‐depleted 4‐week‐old female mice. (E) The expression of GFP and MVH in ovarian tissue from *Stra8‐CreERT2; mT/mG* mice was examined by immunostaining. No GFP (green, arrows) and MVH (red, arrows) double positive germ cells were observed in the oocyte‐depleted ovaries of *Stra8‐CreER* ^*T2*^ *; mT/mG* mice after chemotherapy treatment at 4 weeks. (F) Quantitative analysis of MVH and GFP double positive germ cells in the oocyte‐depleted ovaries of *Stra8‐CreER* ^*T2*^ *; mT/mG* mice at 4 weeks after chemotherapy treatment. For B, C, E and F, *n* = 3.

### 2.2 Generation of an Uninterrupted Lineage Tracing System to Label Meiotic Germ Cells in the Ovaries of Adult Mice

To further continuously trace the *Stra8*‐expressing germ cells in the ovaries of adult females, we first generated a *Stra8‐CrexER* mouse model. The knock‐in strategy and genotyping are illustrated in Figure S3A,B. As shown in Figure 2A, the ERT2 element in *Stra8‐CrexER* ^*T2*^ mice is flanked with two rox sites. Under normal physiological conditions, the activity of *Stra8‐Cre* is dependent on Tamoxifen administration in the *Stra8‐CreER* ^*T2*^ mouse model. By contrast, the ERT2 element will be removed when Dre is activated, and *Stra8‐Cre* will be activated independent of Tamoxifen induction afterwards in the *Stra8‐CrexER* ^*T2*^ mouse model. Additionally, we also generated an *H11‐TLR* reporter mouse model, and the genotyping is illustrated in Figure S4B. As shown in Figure S4, two stop cassettes were inserted between two loxP and rox sites, respectively. Upon Cre‐loxP and Dre‐rox mediated recombination, the expression of ZsGreen or tdTomato is activated. Meanwhile, the Cre‐loxP and Dre‐rox recombination are mutually independent and do not influence each other.

**FIGURE 2:**
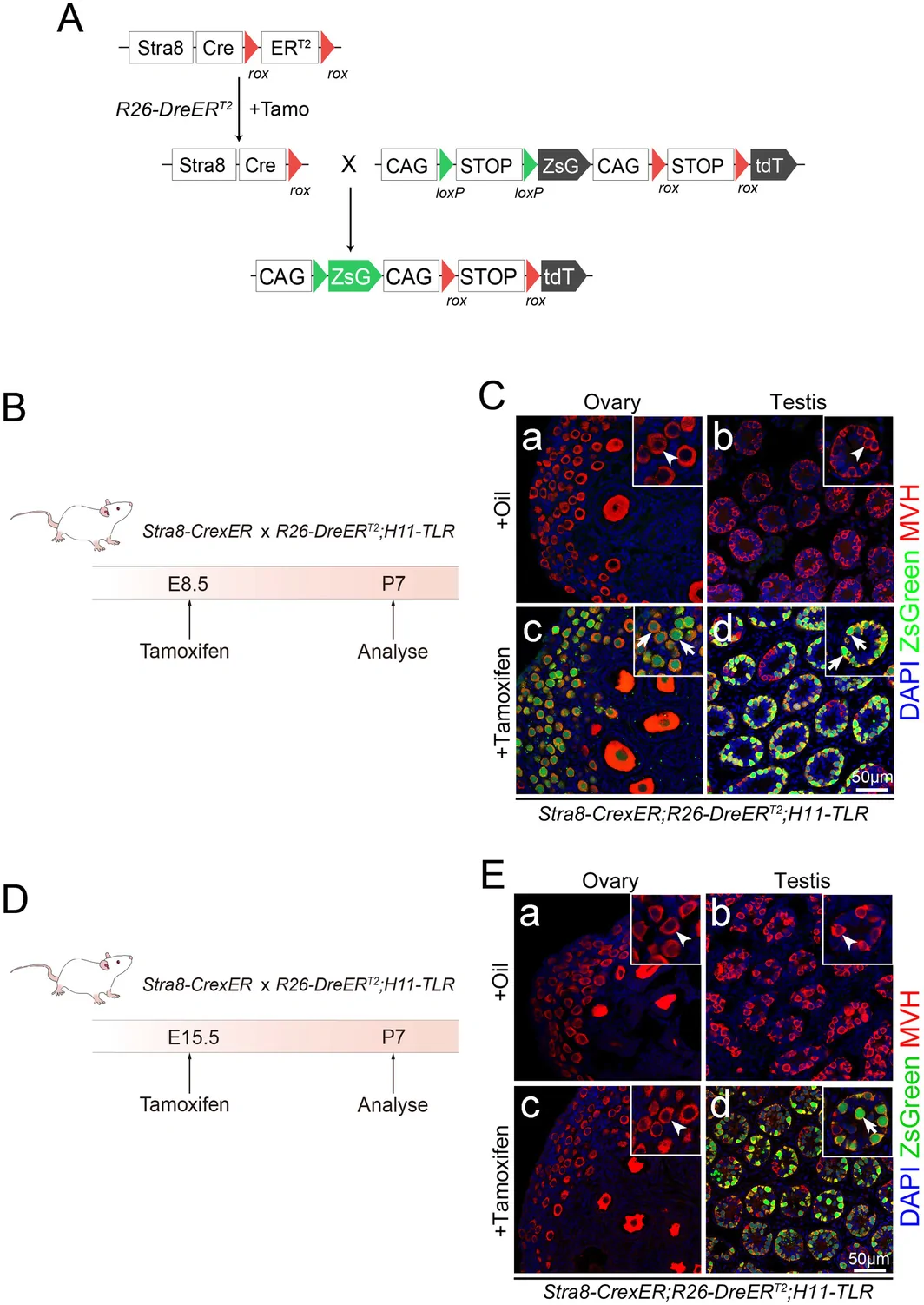
Generation and characterisation of seamless lineage tracing mouse model. (A) Schematic illustration of long‐term uninterrupted lineage tracing strategy. In this mouse model, *Stra8‐CreER* ^*T2*^ was switched into *Stra8‐Cre* after removing the ERT2 element by Tamoxifen‐induced Dre. (B) Schematic illustration of experimental strategy to verify the mouse model. (C) *Stra8‐CrexER; R26‐DreER* ^*T2*^ *; H11‐TLR* mice were treated with Tamoxifen at E8.5 and the expression of ZsGreen (green) and MVH (red) in germ cells was examined by immunostaining at P7. The expression of ZsGreen (white arrows) was detected in both male and female germ cells (white arrows) at P7 with Tamoxifen treatment. (D) Schematic illustration of experimental strategy to verify the mouse model. (E) *Stra8‐CrexER; R26‐DreER* ^*T2*^ *; H11‐TLR* mice were treated with Tamoxifen at E15.5 and the expression of ZsGreen (green) and MVH (red) in germ cells was examined by immunostaining at P7. The expression of ZsGreen was detected in male germ cells (whitearrow), but not in female germ cells at P7 with Tamoxifen treatment (white arrowhead). For B, C, D and E, *n* = 3.

To verify the mouse model, *Stra8‐CrexER* mice were crossed with *H11‐TLR* reporter mice and *R26‐DreER* ^*T2*^ mice. The pregnant females were treated with Tamoxifen or sesame oil at E8.5 and ovaries and testes were collected at P7 (Figure 2B). ZsGreen signal was detected in germ cells of both male and female *Stra8‐CrexER; R26‐DreER* ^*T2*^ *; H11‐TLR* mice with Tamoxifen treatment (Figure 2C, white arrows). No ZsGreen signal was detected in other tissues, such as heart, kidney, liver, lung and brain in *Stra8‐CrexER; R26‐DreER* ^*T2*^ *; H11‐TLR* or *R26‐DreER* ^*T2*^ *; H11‐TLR* mice(Figure S5), indicating that *Stra8‐CrexER* was switched into *Stra8‐Cre* with Tamoxifen treatment before E12.5 and the system possessed no leaky expression. To further verify this mouse model, pregnant females were treated with Tamoxifen at E15.5 and the testes and ovaries were collected at P7 for analysis (Figure 2D). ZsGreen‐positive germ cells were observed in the testes, but not in the ovaries of *Stra8‐CrexER; R26‐DreER* ^*T2*^ *; H11‐TLR* mice (Figure 2E). Additionally, pregnant females were treated with Tamoxifen or sesame oil at E8.5 and the testes and ovaries were analysed at E17.5. ZsGreen and MVH double positive germ cells were only detected in the ovaries with Tamoxifen administration (Figure S3C,D). These results indicated that the activity of *Stra8‐Cre* could be induced uninterruptedly with Tamoxifen treatment in *Stra8‐CrexER; R26‐DreER* ^*T2*^ *; H11‐TLR* mice and the system ensures the precise activation at appropriate time.

### 2.3 No Stra8‐Expressing Germ Cells Were Detected in Ovaries After Birth With Uninterrupted Lineage Tracing

To further determine whether new oocytes are generated through meiosis in postnatal ovaries, female *Stra8‐CrexER; R26‐DreER* ^*T2*^ *; H11‐TLR* mice were treated with Tamoxifen at 4 weeks. The ovaries were collected at 3 and 6 months post‐treatment for further analysis (Figure 3A). Serial sections of ovaries were prepared and the expression of ZsGreen and MVH in all sections was examined by immunostaining. The results of immunostaining showed that all the germ cells were labelled with MVH, but no ZsGreen signal was detected at both 3 and 6 months after Tamoxifen treatment(Figure 3B,D). The proportion of ZsGreen and MVH double positive germ cells was quantitatively analysed across serial sections. The results showed that all the MVH‐positive germ cells were ZsGreen negative and no ZsGreen and MVH double positive germ cells were detected at both 3 and 6 months after Tamoxifen treatment (Figure 3C,E). These results indicated that no germ cells initiate meiosis in adult mouse ovaries under physiological conditions.

**FIGURE 3:**
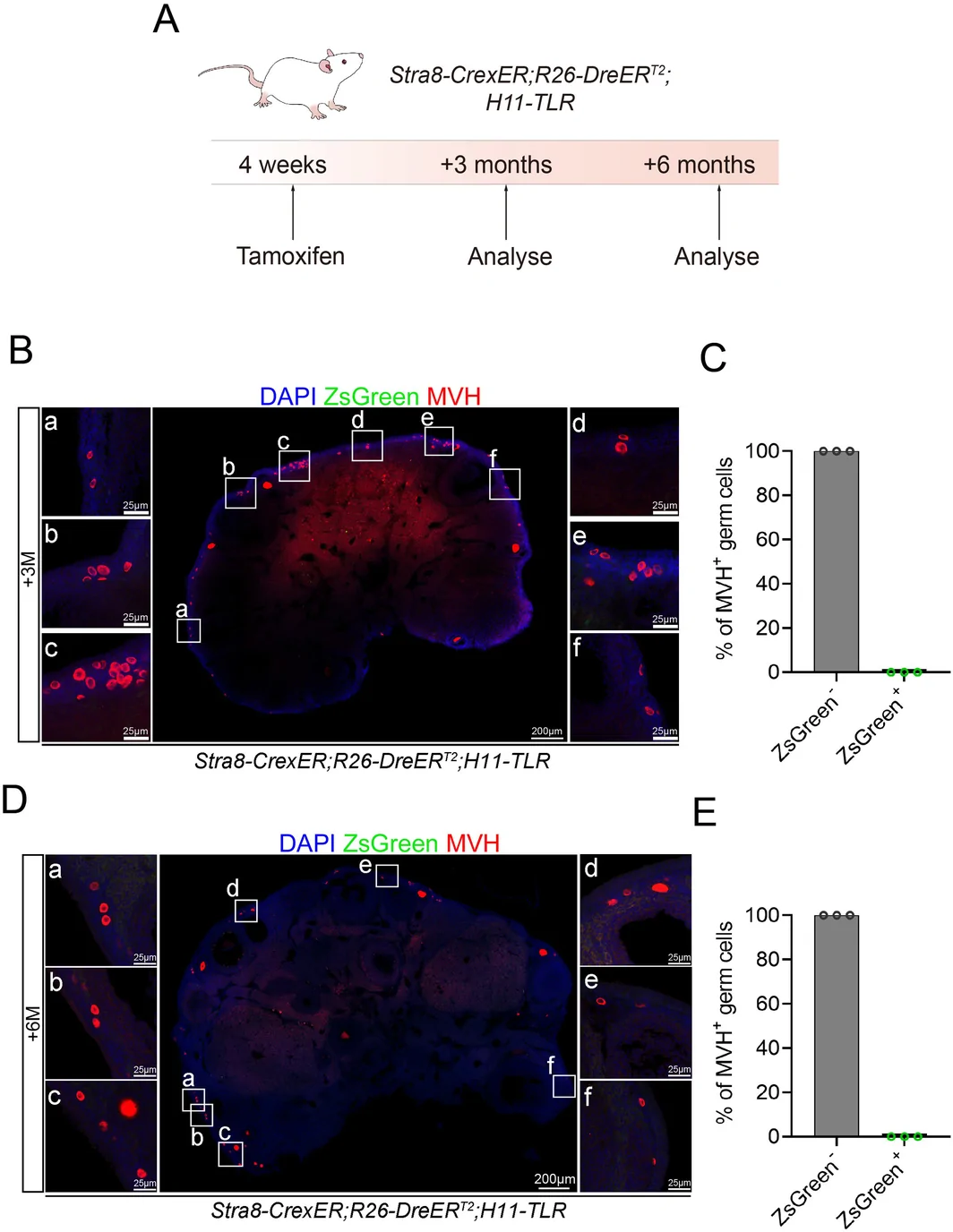
No Stra8‐expressing germ cells were detected in the ovaries of female mice after 3 and 6 months with continuous and seamless lineage tracing system. (A) Schematic illustration of the experimental strategy to label the Stra8‐positive meiotic cells in adult female mice using the *Stra8‐CrexER; R26‐DreER* ^*T2*^ *; H11‐TLR* mouse model. Female *Stra8‐CrexER; R26‐DreER* ^*T2*^ *; H11‐TLR* mice were treated with tamoxifen at 4 weeks and the expression of ZsGreen and MVH in germ cells was examined at 3 and 6 months after tamoxifen treatment. (B) The expression of ZsGreen and MVH in the ovaries of *Stra8‐CrexER; R26‐DreER* ^*T2*^ *; H11‐TLR* mice was examined by immunostaining. No ZsGreen (green) and MVH (red) double‐positive germ cells were observed in the ovaries of *Stra8‐CrexER; R26‐DreER* ^*T2*^ *; H11‐TLR* at 3 months after tamoxifen treatment at 4 weeks. (C) Quantitative analysis of MVH and ZsGreen double positive germ cells in the ovaries of *Stra8‐CrexER; R26‐DreER* ^*T2*^ *; H11‐TLR* mice 3 months after tamoxifen treatment. (D) The expression of ZsGreen and MVH in the ovaries of *Stra8‐CrexER; R26‐DreER* ^*T2*^ *; H11‐TLR* mice was examined by immunostaining. No ZsGreen (green) and MVH (red) double positive germ cells were observed in the ovaries of *Stra8‐CrexER; R26‐DreER* ^*T2*^ *; H11‐TLR* at 6 months after tamoxifen treatment. (E) Quantitative analysis of MVH and ZsGreen double positive germ cells in the ovaries of *Stra8‐CrexER; R26‐DreER* ^*T2*^ *; H11‐TLR* mice 6 months after tamoxifen treatment. For B, C, D and E, *n* = 3.

To test whether new oocytes were formed under pathological conditions, *Stra8‐CrexER; R26‐DreER* ^*T2*^ *; H11‐TLR* mice were treated with busulfan and cyclophosphamide after Tamoxifen treatment. The ovaries were collected at 3 and 6 months after busulfan and cyclophosphamide treatment (Figure 5A). We found that the number of germ cells was dramatically reduced after chemotherapy treatment, indicating the exposure of chemotherapy drugs leads to the depletion of oocytes. The results of immunostaining for ZsGreen and MVH revealed that no ZsGreen positive germ cells were observed in the ovaries of *Stra8‐CrexER; R26‐DreER* ^*T2*^ *; H11‐TLR* mice at both 3 and 6 months after busulfan and cyclophosphamide treatment (Figure 5B).

### 2.4 No Other Somatic Cells Contributed to the Pool of Oocyte in Postnatal Female Mice

The results of the above study were obtained by tracing putative fGSCs in adult female mice based on *Stra8* expression during the initiation of meiosis. To further determine whether other cell types contribute to the pool of oocytes in female mice after birth, a dual recombinase‐based lineage tracing system was used. As shown in Figure 4A, *R26‐IR1* reporter mouse model features interleaved loxP sites and rox sites. Specifically, when Cre‐loxP recombination occurs, one rox site is removed, thereby preventing potential Dre‐rox recombination. Thus, this cell population permits only one type of recombination, ensuring exclusive labelling of a specific cell population and preventing further labelling by another recombination system.

**FIGURE 4:**
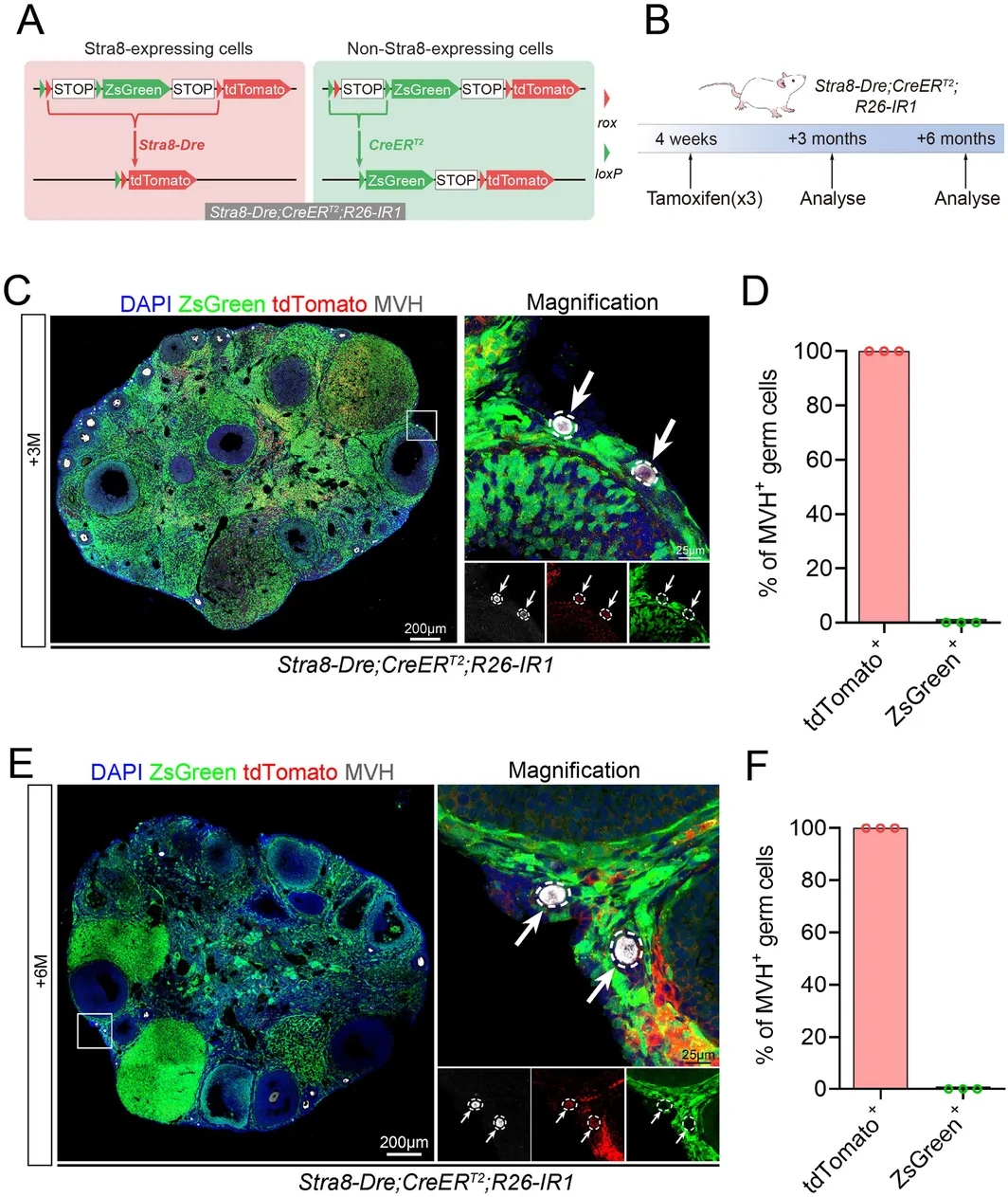
No new oocytes were formed in the ovaries of adult mice under physiological conditions. (A) Working model for *Stra8‐Dre; CreER* ^*T2*^ *; R26‐IR1* mice. (B) Experimental strategy to test for the generation of ZsGreen‐positive germ cells in adult female *Stra8‐Dre; CreER* ^*T2*^ *; R26‐IR1* mice. Female *Stra8‐Dre; CreER* ^*T2*^ *; R26‐IR1* mice were treated with tamoxifen for 3 consecutive times at 4 weeks and the expression of ZsGreen, tdTomato and MVH in ovaries was examined by immunostaining at 3 and 6 months after tamoxifen treatment. (C) The expression of ZsGreen, tdTomato and MVH in ovaries from *Stra8‐Dre; CreER* ^*T2*^ *; R26‐IR1* mice at 3 months after tamoxifen treatment was examined by immunostaining. All the germ cells were labelled with MVH (grey, white arrows) and tdTomato (red, white arrows). No ZsGreen (green) positive germ cells were observed in the ovaries of *Stra8‐Dre; CreER* ^*T2*^ *; R26‐IR1* mice. (D) Quantitative analysis of MVH/tdTomato and MVH/ZsGreen double positive germ cells in the ovaries of *Stra8‐Dre; CreER* ^*T2*^ *; R26‐IR1* mice at 3 months after tamoxifen treatment. (E) The expression of ZsGreen, tdTomato and MVH in ovaries from *Stra8‐Dre; CreER* ^*T2*^ *; R26‐IR1* mice at 6 months after Tamoxifen treatment was examined by immunostaining. All the germ cells were labelled with tdTomato (red) and MVH (grey, white arrows). No ZsGreen (green) positive germ cells were observed in the ovaries of *Stra8‐Dre; CreER* ^*T2*^ *; R26‐IR1* mice. (F) Quantitative analysis of MVH/tdTomato and MVH/ZsGreen double positive germ cells in the ovaries of *Stra8‐Dre; CreER* ^*T2*^ *; R26‐IR1* mice at 6 months after Tamoxifen treatment. For C, D, E and F, *n* = 3.

To employ the dual recombinase‐based lineage tracing system, we first generated a *Stra8‐Dre* knock‐in mouse model. The strategy for construction was shown in Figure S6A. Dre was inserted at the upstream of the stop codon in the last exon of the *Stra8* gene. The genotype for *Stra8‐Dre* mice was verified with primers as illustrated in Figure S6B. *Stra8‐Dre* mouse model was verified by crossing with *R26‐IR1* reporter mice. All germ cells in both male and female *Stra8‐Dre; R26‐IR1* testes and ovaries were tdTomato positive at P8, whereas no tdTomato signal was detected in control mice (Figure S6C,D). tdTomato signal was also undetectable in other tissues (such as heart, liver, lung, kidney and brain) of *Stra8‐Dre; R26‐IR1* mice (Figure S6E). These results indicated that *Stra8‐Dre* could be specifically activated in germ cells undergoing meiosis.

To test whether other cells could contribute to the oocyte pool in adult mice, 4‐week‐old *Stra8‐Dre; CreER* ^*T2*^ *; R26‐IR1* female mice were treated with Tamoxifen and ovaries were collected at 3 or 6 months after induction (Figure 4B). In this system, pre‐existing oocytes could be labelled with tdTomato mediated by *Stra8‐Dre* and all the rest cells will be labelled with ZsGreen by *CreER* ^*T2*^ after Tamoxifen induction. Serial sections of ovaries were prepared and all sections were analysed by immunostaining. With Tamoxifen treatment, strong ZsGreen^+^ signal was detected throughout the entire ovaries coupled with the tdTomato signal of MVH‐positive germ cells. All the MVH‐positive germ cells were labelled with tdTomato, but no ZsGreen and MVH double positive germ cells were detected at both 3 and 6 months after Tamoxifen induction(Figure 4C,E). The results of quantitative analysis also showed that all germ cells were tdTomato positive and no ZsGreen positive germ cells were detected in *Stra8‐Dre; CreER* ^*T2*^ *; R26‐IR1* female mice at both 3 and 6 months after Tamoxifen induction (Figure 4D,F).

To test whether new oocytes were formed under pathological conditions, *Stra8‐Dre; CreER* ^*T2*^ *; R26‐IR1* female mice were treated with busulfan and cyclophosphamide after Tamoxifen administration. The ovaries were collected at 3 and 6 months after chemotherapy treatment (Figure 5C). We found that the number of germ cells was dramatically reduced after chemotherapy treatment, indicating that exposure to chemotherapy drugs leads to the depletion of oocytes. The results of immunostaining for ZsGreen, tdTomato and MVH revealed that no ZsGreen positive germ cells were observed at both 3 and 6 months after chemotherapy drug administration (Figure 5D). All these results indicated that no other cell type contributes to the oocyte pool in postnatal female mice under both physiological and pathological conditions.

**FIGURE 5:**
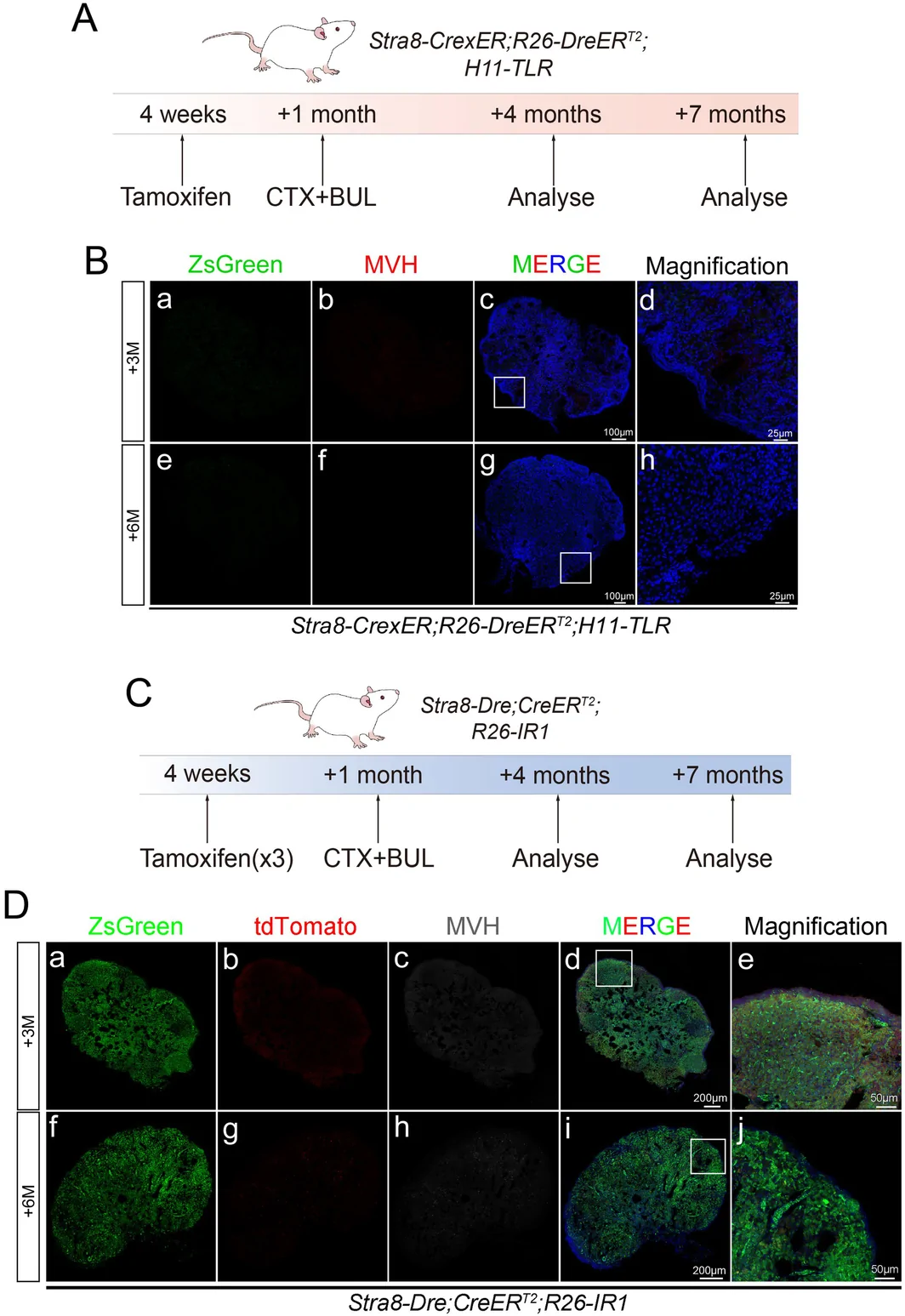
No new oocytes were formed in the ovaries of adult mice under pathological conditions. (A) Schematic illustration of the tracing strategy using *Stra8‐CrexER; R26‐DreER* ^*T2*^ *; H11‐TLR* mouse model under pathological conditions. Female *Stra8‐CrexER; R26‐DreER* ^*T2*^ *; H11‐TLR* mice were treated with Tamoxifen at 4 weeks and the germ cells were depleted with busulfan and cyclophosphamide treatment 1 month later. (B) The expression of ZsGreen and MVH in ovaries from *Stra8‐CrexER; R26‐DreER* ^*T2*^ *; H11‐TLR* mice was examined by immunostaining. No MVH positive germ cells were observed in the ovaries of *Stra8‐CrexER; R26‐DreER* ^*T2*^ *; H11‐TLR* at 3 and 6 months after chemotherapy treatment. (C) Schematic illustration of tracing strategy using *Stra8‐Dre; CreER* ^*T2*^ *; R26‐IR1* mice under pathological conditions. Female *Stra8‐Dre; CreER* ^*T2*^ *; R26‐IR1* mice were treated with Tamoxifen at 4 weeks and the germ cells were depleted by treatment with busulfan and cyclophosphamide 1 month later. (D) The expression of ZsGreen, tdTomato and MVH in ovaries from *Stra8‐Dre; CreER* ^*T2*^ *; R26‐IR1* mice was examined by immunostaining. No MVH positive germ cells were observed in the ovaries of *Stra8‐Dre; CreER* ^*T2*^ *; R26‐IR1* mice at 3 and 6 months after chemotherapy treatment. For B and D, *n* = 3.

## 3 Discussion and Conclusion

In mammals, the pool of primordial follicles is formed perinatally through the process of cyst breakdown. The primordial follicles possess three distinct developmental fates: (1) remaining quiescent; (2) undergoing atresia in quiescence; (3) being activated through initial recruitment [5]. Traditionally, it is believed that the number of primordial follicles is fixed once formed perinatally and it will not be replenished thereafter. However, in recent years, the existence of female germline stem cells (fGSCs) in postnatal ovaries has become a topic of intense debate.

Besides the evidence demonstrating that fGSCs probably reside in bone marrow and peripheral blood [17], opposite results are obtained by other research groups [20, 21, 23, 24, 25]. Especially, Eggan et al.'s study using a parabiosis model refutes the bone marrow origin hypothesis [20]. Wu's group successfully isolated MVH‐positive fGSCs and demonstrated that these cells could be amplified under in vitro condition. Most importantly, the offspring were obtained after fGSCs transplantation [10]. However, these results could not be repeated by other research groups [22]. Although the fGSCs notion has also been tested by other groups, the results remain controversial. Based on these results, it is still hard to draw a conclusion on whether fGSCs exist in adult females.

Cell lineage tracing experiment is the most reliable approach to map cell fate during development. This strategy has been widely used in numerous studies [30, 31, 36, 37, 38]; however, no direct evidence has been obtained due to the lack of a specific marker and the limitations of the labelling system. Conventional lineage tracing systems could not directly label the cells which potentially develop into oocytes in adult females. In mammals, female germ cells are originated from primordial germ cells (PGCs) and initiate meiosis right after sex determination. As a gatekeeper of meiosis initiation, the expression of *Stra8* is directly stimulated by retinoic acid (RA) and first detected in germ cells of female mice at E12.5 [33, 34, 35]. Its expression is absent in female germ cells after E15.5 [35]. In male mice, *Stra8* starts to express in germ cells approximately 5 days after birth and is continuously expressed in the premeiotic germ cells of puberty and adult testes [39]. Theoretically, *Stra8* will not be expressed in female germ cells after birth. If fGSCs exist and new oocytes are formed in adult females, they must undergo meiosis and the expression of *Stra8* could be detected in adult ovaries.

In 2017, Wang et al. used the *Stra8‐rtTA; tetO‐Cre; Rosa26‐YFP* mouse model to label Stra8‐expressing germ cells in adult female mice by treating the mice with doxycycline for 21 days. Interestingly, YFP^+^ germ cells were observed in the ovaries of *Stra8‐rtTA; tetO‐Cre; Rosa26‐YFP* mice, and YFP^+^ offspring were obtained by crossing with wild‐type males [19]. In this study, we used *Stra8‐CreER* ^*T2*^ and *mT‐mG* reporter mouse models to trace germ cells which were undergoing meiosis in adult females. In this model, the *Stra8‐Cre* was activated upon Tamoxifen induction. To test whether *Stra8*‐expressing germ cells exist in adult ovaries, *Stra8‐CreER* ^*T2*^ *; mT‐mG* mice were injected with Tamoxifen at 4 weeks old. To improve the labelling efficiency, the mice were injected with Tamoxifen for six consecutive times. However, no GFP positive germ cells were detected in the ovaries 1 week after Tamoxifen induction, which was inconsistent with Wang et al.'s study [19]. The limitation of this model is that Cre activity is temporarily activated upon Tamoxifen treatment. To continuously trace the *Stra8*‐expressing germ cells in the ovaries after a certain developmental stage, we generated a *Stra8‐CrexER* mouse model, and the *Stra8‐Cre* can be permanently activated upon Tamoxifen‐induced Dre activity. This strategy has previously been used to trace hepatocyte proliferation during liver homeostasis and regeneration [31]. We traced the *Stra8*‐expressing germ cells up to 6 months, and more than 8000 germ cells from three individual mice were examined. However, no ZsGreen positive germ cells were detected. Theoretically, compared to previous study [18, 19, 23, 24, 25], the tracing system used in our study was more reliable.

The advantage of dual recombinase‐based interleaved reporter lineage tracing system(*R26‐IR1*) is simultaneously labelling the target cells and all the rest of cells with different fluorescence. This system minimises the interference of unintended labelling while ensuring accurate activation of the appropriate cell types at the designated time point. In this system, the pre‐existing oocytes were labelled with *Stra8‐Dre* during embryonic stage and all the rest of non‐germ cells were labelled with *CreER* ^*T2*^ at 4 weeks by Tamoxifen induction. The efficiency of *CreER* ^*T2*^ is dependent on the dosage of Tamoxifen. In this study, more than 90% of non‐germ cells were labelled with ZsGreen and no oocyte was ZsGreen positive, indicating that non‐germ cells were efficiently labelled with *CreER* ^*T2*^ and the loxP sites in all the pre‐existing oocytes were specifically blocked by *Stra8‐Dre*. More than 8000 oocytes from 3 individual mice were examined and the cells were traced up to 6 months. Moreover, we examined this lineage‐tracing system at both physiological and pathological conditions. Theoretically, if fGSCs exist and contribute to the pool of oocyte after birth, it must be detected by our tracing system. In this model, the germ cells were labelled with MVH, which is a general germ cell marker.

The *Stra8*‐based lineage‐tracing strategy allowed us to trace whether fGSCs exist and contribute to oocytes through meiosis. However, this strategy alone may not be sufficient to rule out the possibility that somatic cells could give rise to germ cells without undergoing meiosis. To further investigate this possibility, the mutually exclusive lineage‐tracing system (*Stra8‐Dre; CreER* ^*T2*^ *; R26‐IR1*) was used to label germ cells and non‐germ cells independently. With long‐term tracing, this system enabled us to detect the *de novo* oogenesis whether meiosis was initiated or not in postnatal mice. In summary, no fGSCs were detected in adult female mice under both physiological and pathological conditions using advanced dual recombinase‐based lineage tracing systems. Our findings provide direct evidence against the existence of fGSCs in female mice, and suggest caution against the premature proposal of clinical applications. In this study, we investigated the existence of fGSCs using genetically modified mouse models. Whether other mammalian species contain fGSCs needs further investigation.

## 4 Methods

### 4.1 Mice

All mouse experiments were performed in accordance of Institutional Animal Care and Use Committee (IACUC) of the Institute of Zoology, Chinese Academy of Sciences (IOZ‐IACUC‐2024‐184). The mice used in this study were of mixed background consisting of C57BL/6J and ICR. The generation of *mT/mG, Cre‐ER* ^*T2*^, *R26‐DreER* ^*T2*^ and *R26‐IR1* was as described previously [40, 41, 42]. *Stra8‐CreER* ^*T2*^, *Stra8‐CrexER*, *Stra8‐Dre* and *H11‐TLR* mouse models were generated using CRISPR‐Cas9 technology. *Stra8‐CreER* ^*T2*^, *Stra8‐CrexER* and *Stra8‐Dre* mouse models were generated by the Animal Experiment Center, Institute of Zoology. *H11‐TLR* mouse model was generated using CRISPR‐Cas9 technology by Shanghai Biomodel Organism. The primers used for genotyping are listed in Table S1.

For induction of CreER‐loxP or DreER‐rox recombination, tamoxifen (Sigma, T5648) or 4‐hydroxytamoxifen (Sigma, H6278) was dissolved in sesame oil and administered by oral gavage at the indicated time points at doses of 0.1–0.25 mg/g body weight for tamoxifen and 60 μg/g body weight for 4‐hydroxytamoxifen, as described previously [31, 43]. To establish the chemotherapy‐induced premature ovarian insufficiency (POI) model, mice were administered 120 mg/kg cyclophosphamide (CTX, Sigma, C0768) and 12 mg/kg busulfan (BUS, Sigma, B2635) at the indicated time points via intraperitoneal injection.

### 4.2 Immunostaining

Immunostaining was performed according to previous protocols [31, 40]. Briefly, ovaries and other organs were collected and washed in PBS to remove excessive blood, and then fixed with 4% paraformaldehyde at 4°C. The fixed tissues were dehydrated in both 10% and 25% sucrose solutions in PBS at 4°C until tissues were fully penetrated. The tissues were then embedded in optimum cutting tissue (O.C.T., Sakura) and stored at −80°C. Cryosections with a thickness of 20 μm were collected with slides, washed in PBS, and incubated with blocking solution containing 5% bovine serum albumin (BSA) and 0.3% Triton X‐100 for 1 h at room temperature. Sections were then incubated with primary antibodies diluted in 1% BSA for 1 h at room temperature or overnight at 4°C. After washing three times in PBS, tissue sections were incubated with Alexa Fluor‐conjugated secondary antibodies and DAPI for 1 h at room temperature. After that, slides were washed with PBS and mounted with fluorescence aqueous mounting medium (Sigma, F4680). For immunofluorescence (IF) analysis of paraffin sections, 5 μm paraffin sections were deparaffinised and rehydrated. Following antigen retrieval, the staining and incubation procedures for paraffin sections were similar to those for cryosections. The antibodies employed were as follows: anti‐GFP (1:1000, Abcam, ab13970), anti‐MVH (1:200, Abcam, ab13840) and anti‐STRA8 (1:200, Abcam, ab308124).

For paraffin sections, ovaries were serially sectioned into 5‐μm thickness, and one out of every five consecutive sections was examined throughout the ovary. For frozen sections, all sections obtained by serially sectioning were analysed. To avoid repeated counting, oocytes were counted only when a clearly identified nucleus was present. Multiple sections from the same mouse were analysed, but individual sections were not considered independent biological replicates.

### 4.3 Statistical Analysis

All mice of each genotype were randomly assigned to different groups, and all experiments were performed using at least three individual animals. Statistical analyses were performed using GraphPad Prism version 9.5.1.

## Author Contributions

**Fei Gao**, **Hongmei Wang** and **Kui Liu:** conceptualisation. **Min Chen** and **Xiuhong Cui:** methodology. **Yang Gao**, **Zhiming Shen** and **Mengyue Wang:** investigation. **Yang Gao** and **Zhiming Shen:** visualisation. **Yang Gao** and **Fei Gao:** writing – original draft. **Fei Gao**, **Hongmei Wang**, **Kui Liu** and **Mengyue Wang:** writing – review and editing. **Fei Gao**, **Hongmei Wang**, **Kui Liu** and **Mengyue Wang:** supervision. All co‐authors read and approved the manuscript.

## Funding

This work was supported by the National Natural Science Foundation of China (82421003, 32530031); Strategic Priority Research Program of the Chinese Academy of Sciences (XDB0820000) and the National Natural Science Foundation of China (32270902, 32170855, 32601144, 32300678).

## Conflicts of Interest

The authors declare no conflicts of interest.

## Supporting information


**Figure S1:** Generation of *Stra8‐CreER* ^*T2*^ mouse model.
**Figure S2:** Characterisation of *Stra8‐CreER* ^*T2*^ mouse model.
**Figure S3:** Generation and characterisation of *Stra8‐CrexER* mouse model.
**Figure S4:** Generation and characterisation of *H11‐TLR* mouse model.
**Figure S5:** No ZsGreen positive cells were observed in other organs of *Stra8‐CrexER; R26‐DreER* ^*T2*^ *; H11‐TLR* and *R26‐DreER* ^*T2*^ *; H11‐TLR* mice after Tamoxifen treatment.
**Figure S6:** Generation and characterisation of *Stra8‐Dre* mouse model.
**Table S1:** The primers for genotyping.

## Data Availability

The data that support the findings of this study are available from the corresponding author upon reasonable request.
