# Supplementary material for: No Female Germline Stem Cells Were Identified by Advanced Dual Recombinase‐Based Lineage Tracing System
Source: Cell Prolif. 2026 Oct 20:e70289. Online ahead of print. doi: 10.1111/cpr.70289 (PMC13682860; doi:10.1111/cpr.70289)
Supplement: Supplementary file 1 [file CPR-9999-e70289-s001.docx]

**No female germline stem cells were identified in mice by advanced dual recombinase-based lineage tracing system**

**This PDF file includes:**

Figs. S1-S6

Tables S1

**Supplementary figures**

**Figure S1. Generation of *Stra8-CreER^T2^* mouse model.**

A. Strategy for generation of *Stra8-CreER^T2^* knock-in alleles using CRISPR-Cas9 system.
B. The genotype of *Stra8-CreER^T2^* mice was verified by PCR.

**Fig S2. Characterization of *Stra8-CreER^T2^* mouse model.**

A, C and E. Schematic illustration of experimental strategy to verify the mouse model.

B. *Stra8-CreER^T2^; mT-mG* mice were treated with Tamoxifen at E12.5 and the expression of GFP (green) in STRA8-positive cells (red) was examined by immunostaining at E14.5. The expression of GFP was detected in female germ cells at E14.5 with Tamoxifen treatment(white arrows).

D. *Stra8-CreERT2; mT-mG* mice were treated with Tamoxifen at E15.5 and the expression of GFP (green)and in MVH-positive germ cells (red) was examined by immunostaining at P7. No GFP signal was detected in female germ cells at P7 with Tamoxifen treatment (white arrowhead).

F. 4-week-old *Stra8-CreERT2; mT-mG* mice were treated with Tamoxifen. The expression of GFP (green) was examined by immunostaining in STRA8-positive cells (red) in the testes and in MVH-positive germ cells(red) in the ovaries 2 days later. GFP signal was detected in male germ cells with Tamoxifen treatment (white arrow).

For B, D and F, n = 3.

**Figure S3. Generation and characterization of *Stra8-CrexER* mouse model.**

A. Schematic illustration of the strategy for generating *Stra8-CrexER* mouse model.

B. The genotype of *Stra8-CrexER* mice was verified by PCR.

C. Schematic illustration of experimental strategy to verify the mouse model. *Stra8-CrexER* mice were crossed with *R26-DreER^T2^; H11-TLR* mice. The pregnant females were treated with Tamoxifen at E8.5 and the expression of ZsGreen in germ cells was examined by immunostaining at E17.5.

D. No ZsGreen signal was detected in germ cells of both male (white arrowhead) and female (white arrowhead) *Stra8-CrexER; R26-DreER; H11-TLR* mice with sesame oil treatment. ZsGreen signal was detected in female germ cells (white arrows) with Tamoxifen, but not in male germ cells (white arrowhead). For D, n = 3

**Figure S4. Generation and characterization of *H11-TLR* mouse model.**

A. Schematic illustration of the strategy for generating *H11-TLR* mouse model.

B. The genotype of *H11-TLR* mice was verified by PCR.

**Figure S5. No ZsGreen positive cells were observed in other organs of *Stra8-CrexER; R26-DreER^T2^; H11-TLR* and *R26-DreER^T2^; H11-TLR* mice after Tamoxifen treatment.**

A. The expression of ZsGreen (green) in different organs of S*tra8-CrexER; R26-DreER^T2^; H11-TLR* mice was examined. No ZsGreen signals were observed in the heart, kidney, liver, lung and brain with or without tamoxifen treatment.

B. The expression of ZsGreen (green) in different organs of *R26-DreER^T2^; H11-TLR* mice was examined. No ZsGreen signals were observed in the heart, kidney, liver, lung and brain with or without tamoxifen treatment. For A and B, n = 3.

**Figure S6. Generation and characterization of *Stra8-Dre* mouse model.**

A. Schematic strategy for generation of *Stra8-Dre* knock-in alleles by CRISPR-Cas9 system.

B. The genotype of *Stra8-Dre* mice was verified by PCR.

C. The expression of tdTomato (red) and MVH (green) in ovaries and testes from *Stra8-Dre; R26-IR1* mice was examined by immunostaining. No tdTomato signal was detected in MVH positive germ cells of both testes and ovaries in *R26-IR1* mice. tdTomato signal was detected in MVH positive germ cells of both testes and ovaries in *Stra8-Dre; R26-IR1* mice.

D. Quantitative analysis of MVH and tdTomato double positive germ cells in the ovaries of *Stra8-Dre; R26-IR1* mice.

E. No tdTomato (red) signal was detected in other organs of *Stra8-Dre; R26-IR1* mice.

For C, D and E, n = 3

| **The primers for genotyping** | | |
| --- | --- | --- |
| Mice | Name | Sequence (5’ to 3’) |
| *Stra8-CreER^T2^* | Stra8-CreERT2-F | AAGCCAATTCTTTGCTGCCC |
|  | Stra8-CreERT2-R | CTTTTGGAAGCAGCCTTTCTCA |
| *Stra8-CrexER* | Stra8-CrexER-F | AAGCCAATTCTTTGCTGCCC |
|  | Stra8-CrexER-R | CTTTTGGAAGCAGCCTTTCTCA |
| *Stra8-Dre* | Stra8-Dre-F | AAGCCAATTCTTTGCTGCCC |
|  | Stra8-Dre-R | CTTTTGGAAGCAGCCTTTCTCA |
| *H11-TLR* | H11-TLR-F | ACCGCAGCGACGCCAAGAACC |
|  | H11-TLR-R | GCAAGGCAGGCGGCGATGAGT |

Table S1 | The primers for genotyping.
